# Robust COX-2-mediated prostaglandin response may drive arthralgia and bone destruction in patients with chronic inflammation post-chikungunya

**DOI:** 10.1371/journal.pntd.0009115

**Published:** 2021-02-17

**Authors:** Yosra Bedoui, Axelle Septembre-Malaterre, Claude Giry, Marie-Christine Jaffar-Bandjee, Jimmy Selambarom, Pascale Guiraud, Philippe Gasque

**Affiliations:** 1 Unité mixte de recherche sur les processus infectieux en milieu insulaire tropical, INSERM U1187, CNRS 9192, IRD 249, Université de La Réunion—Plateforme Technologique CYROI Sainte-Clotilde, Île de La Réunion, France; 2 Laboratoire d’immunologie clinique et expérimentale de la zone de l’océan indien CHU La Réunion site Félix Guyon, Allée des Topazes, Saint Denis de La Réunion, France; 3 Unité de recherche Etudes Pharmaco-Immunologie, Université de la Réunion, CHU La Réunion site Félix Guyon, Allée des Topazes, Saint Denis de La Réunion, France; 4 Laboratoire de biologie, CNR associé des arbovirus, CHU La Réunion site Félix Guyon, Allée des Topazes, Saint Denis de La Réunion, France; University of Texas Medical Branch, UNITED STATES

## Abstract

Patients following infection by chikungunya virus (CHIKV) can suffer for months to years from arthralgia and arthritis. Interestingly, methotrexate (MTX) a major immune-regulatory drug has proved to be of clinical benefit. We have previously shown that CHIKV can persist in the joint of one patient 18 months post-infection and plausibly driving chronic joint inflammation but through ill-characterized mechanisms. We have pursued our investigations and report novel histological and *in vitro* data arguing for a plausible role of a COX-2-mediated inflammatory response post-CHIKV. In the joint, we found a robust COX-2 staining on endothelial cells, synovial fibroblasts and more prominently on multinucleated giant cells identified as CD11c+ osteoclasts known to be involved in bone destruction. The joint tissue was also strongly stained for CD3, CD8, CD45, CD14, CD68, CD31, CD34, MMP2, and VEGF (but not for NO synthase and two B cell markers). Dendritic cells were rarely detected. Primary human synovial fibroblasts were infected with CHIKV or stimulated either by the synthetic molecule polyriboinosinic:polyribocytidylic acid (PIC) to mimic chronic viral infection or cytokines. First, we found that PIC and CHIKV enhanced mRNA expression of COX-2. We further found that PIC but not CHIKV increased the mRNA levels of cPLA2α and of mPGES-1, two other central enzymes in PGE2 production. IFNβ upregulated cPLA2α and COX-2 transcription levels but failed to modulated mPGES-1 mRNA expression. Moreover, PIC, CHIKV and IFNβ decreased mRNA expression of the PGE2 degrading enzyme 15-PGDH. Interestingly, MTX failed to control the expression of all these enzymes. In sharp contrast, dexamethasone was able to control the capacity of pro-inflammatory cytokines, IL-1β as well as TNFα, to stimulate mRNA levels of cPLA2α, COX-2 and mPGES-1. These original data argue for a concerted action of CHIKV (including viral RNA) and cytokines plausibly released from recruited leukocytes to drive a major COX-2-mediated PGE2 proinflammatory responses to induce viral arthritis.

## Author summary

It is important to have a better understanding of the immuno-pathogenesis of Chikungunya virus (CHIKV) and particularly focusing on the chronic phase associated to arthralgia and arthritis. Benefiting from our prospective cohort studies, we herein provide novel *in vivo* data identifying for the first time the implication of COX-2 and several other enzymes involved in prostaglandin biosynthesis and the persistence of the virus on the joint. Prostaglandin has major activities in inflammation and joint destruction. *In vitro*, we have used a model of human synovial fibroblasts to decipher the regulatory mechanisms of prostaglandin biosynthesis pathway. We have made important observations showing that the virus itself as well as major inflammatory cytokines can dramatically control the expression of all enzymes involved in the metabolism of prostaglandin. Interestingly, pharmacological investigations further revealed that dexamethasone, but not methotrexate (currently used to treat patients with chikungunya) may be of clinical values.

## Introduction

In the recent years, chikungunya virus (CHIKV) has re-emerged as one of the many arthropod-borne viruses that can cause significant public health threats with high social and economic impact [1,2]. CHIKV, which causes chikungunya fever (CHIKF), is an alphavirus belonging to the Togaviridae family. CHIKF is an acute illness with abrupt fever, rash, myalgia, and severe incapacitating arthralgia [3,4]. Although these acute symptoms usually resolve within two weeks, some patients go on to develop persistent and/or recurrent joint pains that may last for months or years after contracting CHIKV [5–7]. The precise mechanisms of the progression of the CHIKV disease (CHIKVD) from acute Chikungunya fever to the chronic phase associated with arthralgia remain poorly understood. However, possible viral persistence in the joints with local inflammatory responses mediated by recruited macrophages and T cells may play a role in the chronic manifestations of CHIKVD [8,9].

The eicosanoid prostaglandin (PG) E2 is one of the most important mediators of inflammation and contributes to several pathogenic features of arthritis such as pain, inflammation and bone destruction. PGE2 is abundantly detected in the synovial fluid of rheumatoid arthritis (RA) patients [10]. Cytokine-activated cells, such as synovial cells, macrophages/monocytes, and chondrocytes, are the primary source of PGE2 in arthritic joints [11]. The proinflammatory cytokines IL-1β and TNFα, which play a pivotal role in initiating and driving RA, are known to enhance PGE2 production [12]. The deleterious role of PGE2 in the pathogenesis of arthritis may occur through several mechanisms. PGE2 contributes to synovial inflammation by increasing local blood flow and vascular permeability and potentiating the effects of mediators such as bradykinin and histamine [13,14]. It mediates pain hypersensitization by lowering the activation threshold of afferent pain nerve endings to pain mediators [13,15]. Furthermore, PGE2 has been shown to modulate cartilage and bone metabolism by stimulating macrophage differentiation into osteoclast and matrix metalloproteinases production [12]. PGE2 is also a potent stimulator of angiogenesis within the proliferating synovium by stimulating synovial fibroblasts to produce vascular endothelial growth factor (VEGF) [16].

PGE2 synthesis is initiated with the release of arachidonic acid (AA) from membrane phospholipids by phospholipase A2 enzymes (PLA2) [17]. Among PLA2 enzymes, the cytosolic PLA2 alpha from Subgroup IV (cPLA2α) is known to have the highest specificity for AA and is believed to play a key role in inflammatory diseases [17]. Serum PLA2 activity was found to be increased in RA patients [18]. Furthermore, cPLA2α- deficient mice were found to be resistant to collagen-induced arthritis, suggesting that cPLA2α may play a pivotal role in arthritis [19].

Following cPLA2α activation, free AA is enzymatically metabolized by cyclooxygenases (COX) into PGH2. COXs are membrane-bound heme containing glycoproteins [17]. COX has two isoforms COX-1 and COX-2. Whereas COX-1 is constitutively expressed in the majority of cells under basal conditions, COX-2 is inducible by proinflammatory stimuli such as IL-1β, TNFα and trauma [20]. PGH2 is a substrate for prostaglandin E synthases (PGES) which produces the more stable prostanoid, PGE2 [17]. Three different forms of the PGES enzymes have been identified, microsomal prostaglandin E synthase-1 (mPGES-1), mPGES-2 and cytosolic prostaglandin E synthase (cPGES) [17]. mPGES-1 is the dominant isoform for PGE2 synthesis. mPGES-1 induction has been shown to be coordinated with COX-2 expression under inflammatory conditions in RA synovium [21].

The overall levels of PGE2 are regulated not only by its synthesis but also by its degradation. The key enzyme responsible for the prostaglandin metabolic inactivation is 15-hydroxyprostaglandin dehydrogenase (15-PGDH). PGE2 is converted by 15-PGDH to an inactive 15-keto PGE2 and is therefore rapidly removed from tissues [22].

PGE2 has been proposed as a mediator of the inflammatory process induced by viruses in the joint [23]. CHIKV has been found to regulate cellular PGE2 expression but through ill-characterized mechanisms [24]. Viral infections are associated with inflammation and with interferon (IFN) production. IFN, the most effective mechanism of the innate immunity against viruses, has been suggested to have a role in propagating the inflammatory effect of viral infections through prostaglandins [25]. Interestingly, IFN induction by CHIKV-infected fibroblasts has been found to correlate with increased cellular prostaglandin production [24]. PGE2 in the joints of CHIK patients may be induced by direct viral stimulation (CHIK viral particles or persistent CHIKV RNA) or regulated indirectly via induced IFN production or inflammatory cytokines secretion such as IL-1β and TNFα known to be produced by recruited macrophages. This interesting hypothesis remains to be tested.

Two types of chronic CHIKV-induced rheumatic disorders have been described [1,26]. Patients may experience chronic musculoskeletal disorders and respond to some extent to symptomatic treatment with analgesics and anti-inflammatory drugs. However, other patients meet the criteria for chronic inflammatory rheumatism (CIR) including RA, which can be severely debilitating [1,26]. Better-targeted drugs are clearly needed to treat post-CHIKV CIR. To date, there are no existing official guidelines for the management of chronic CHIKV arthritis. A variety of disease modifying anti-rheumatic drugs (DMARDs), including chloroquine (CHQ), hydroxychloroquine (HCQ) and sulfasalazine (SSZ) have been used, but methotrexate (MTX) may also be of clinical value [26–29]. However, mechanisms by which MTX exerts its therapeutic effect in post-CHIKV chronic arthralgia and arthritis are poorly understood.

Our aim was to pursue our histological investigations of synovial tissue from patients with chronic CHIKF. Moreover, we further used our *in vitro* model of synovial fibroblast (HSF) to investigate the PGE2 biosynthetic pathway and its metabolism in response to CHIKV infection and to assess the potential pharmacological effects of MTX. To mimic cytoplasmic viral dsRNA generated in the synovium of chronically CHIKV infected patients and as previously reported [30], we used the synthetic analog of viral dsRNA PIC. To mimic leukocyte activation in the joint, we tested the effects of proinflammatory cytokines, IL-1β and TNFα.

## Materials and methods

### Ethics statement

Tissue samples (blood, CSF, placenta, muscle and synovial tissue biopsies) were collected whenever possible from a large cohort of hospitalized patients (PHRC adult chikungunya) infected by CHIKV during the 2005/2006 epidemic in La Reunion Island. The study was approved by the Tours IRB (http://cppouest1.fr/mediawiki/index.php?title=Accueil), France (Agreement number: 2006–10) and patients signed a written and informed consent for participation and the use of biological samples for research use only according to the World Medical Association declaration of Helsinki.

### Cells, virus and reagents

Human synovial fibroblasts (HSF) were obtained from ScienCell Research Laboratory (ScienCell, 4700; Clinisciences). The primary cultures of HSF were maintained in Minimum Essential Medium eagle (MEM eagle, PAN Biotech P0408500) supplemented with 10% of decomplemented fetal bovine serum (PAN Biotech, 3302 P290907) and completed with L-glutamine 2 mM (Biochrom AG, K0282), 100U/mL– 0.1 mg/mL penicillin- streptomycin (PAN Biotech, P0607100), 1 mM sodium pyruvate (PAN Biotech, P0443100) and 0.5 μg/mL fungizone (PAN Biotech, P0601001).

The double-stranded polyribonucleotide PIC (cat. no.27-4732-01) was purchased from Amersham Biosciences. Interleukin-1β (IL-1β; cat. no. 200-01B), Tumor necrosis factor-α (TNFα; cat. no. 300-01A) and Interferon β (IFNβ; cat. no. 300-02BC) were purchased from Peprotech.

A clinical isolate (clone CHIKV 4.2) amplified from a CHIKV-infected serum sample (isolated during the 2006 epidemic) was used after two passages on Vero cells [8].

### Cell culture treatment

HSF were cultured in six-well culture plates and incubated at 37°C in a humid atmosphere with 5% CO_2_. When reaching 80–90% of confluence, cells were infected with CHIKV clone 4.2 in a BSL3 facility or stimulated with the double-stranded PIC (100μg/mL or 10μg/mL), IFNβ (1000U/mL or 100U/ml) or the proinflammatory cytokines IL-1β (1ng/mL) and TNFα (10ng/mL) in the presence or not of MTX or DXM (at the therapeutic concentration of 1μM) for 6h and 24h. We used CHIKV at low multiplicity of infection (MOI) (10^−1^ and 10^−4^). HSF were exposed to extracellular PIC stimulation via addition to the culture medium.

### Biopsy and immunohistochemistry

The synovial biopsy (hygroma) of one the patients was used and the chronic status of the disease was established at least 18 months p.i as persisting pain with relapsing arthralgia in more than one small articulation. Immunohistochemistry of the synovial biopsy was performed as previously described [8]. Paraffin wax embedded sections were dewaxed, heated in citrate buffer (0.01 M; pH 6.0) for antigen retrieval, and incubated for 60 minutes with mouse monoclonal antibodies (mAbs). Primay antibodies used are listed in Table 1.

**Table 1:** List of primary antibodies for immunohistochemistry assay.

Primary antibody	Reference
Mouse mAb anti- Cyclooxygenase-2 (COX-2) clone 4H12	NCL-COX-2 Novocastra
Mouse mAb anti- CD14 clone 7	NCL-CD14-223 Novocastra
Mouse mAb anti- CD11c clone 5D11	NCL-L-CD11c-563 Novocastra
Mouse mAb anti- Matrix Metalloproteinase 2 (MMP2) clone 17B11	NCL-MMP2-507 Novocastra
Mouse mAb anti- Myelin Basic Protein (MBP) clone 7H11	NCL-MBP Novocastra
Mouse mAb anti- Nitric Oxide Synthase-1 (NOS-1) clone NOS-125	NCL-NOS-1 Novocastra
Mouse mAb anti- DEC-205 (CD205) clone 11A10	NCL-L-DEC205 Novocastra
Mouse mAb anti- CD45 clone X16/99	NCL-LCA Novocastra
anti CD3, T cell	LN10, Leica
anti CD8, Cytotoxic T cell	4B11, Leica
anti CD20, B cell	L26, Leica
anti CD79a, B cell	110E4, Leica
anti KI67, proliferating cell	K2, Leica
anti CD31, anti-PECAM1, endothelial cell	1A10, Leica
anti CD34, endothelial cell	Qbend10, Leica
anti VEGF, proangiogenic factor	C1, SC7269, Santa Cruz

The sections were subsequently incubated with a biotinylated anti-mouse secondary antibody (Bio-Rad, Marnes la Coquette, France) followed by a streptavidin-peroxidase complex. The color reaction was developed with diaminobenzidine (DAB) substrate as chromogen. All incubations were carried out at room temperature and the sections were washed with phosphate buffered saline between all steps. Slides were counterstained with hematoxylin and mounted.

### Quantitative real-time RT-PCR (qRT-PCR)

Total RNA was extracted from harvested cell culture using RNeasy Plus Mini Kit (QIAGEN, Cat No 74136) according to the manufacturer’s instructions.

qRT-PCR experiments were performed using the One Step Prime Script Syber Green RT-PCR kit from TAKARA (Cat No RR066A) as described previously [30]. The specific primers used are summarized in Table 2.

**Table 2:** List of primers used for qRT-PCR.

Primer name	Sequence (5’-3’)	GenBank accession	Product size (bp)
Hu GAPDH_377F	GAACGGGAAGCTTGTCATCA	NM_002046.5	473
Hu GAPDH_849R	TGACCTTGCCCACAGCCTTG		
Hu COX-2_479F	TGGCTACAAAAGCTGGGAAG	NM_000963.3	173
Hu COX-2_669R	GGGGATCAGGGATGAACTTT		
Hu mPGES-1_135F	CCAAGTGAGGCTGCGGAAGAA	NM_004878.4	339
Hu mPGES-1_473R	GCTTCCCAGAGGATCTGCAGA		
Hu cPLA2α_624F	CATGCCCAGACCTACGATTT	NM_024420.2	163
Hu cPLA2α_786R	CCCAATATGGCTACCACAGG		
Hu 15-PDGH_903F	TGCTTCAAAGCATGGCATAG	NM_000860.5	103
Hu 15-PDGH_1005R	AACAAAGCCTGGACAAATGG		
Hu IL-1β_177F	ACAGATGAAGTGCTCCTTCCA	NM_000576.2	73
Hu IL-1β_249R	GTCGGAGATTCGTAGCTGGAT		

qRT-PCR was carried out in LightCycler 480 Instrument II from Roche Life Science with the following steps: a reverse transcription at 42°C for 5 minutes and 40 cycles of a denaturation step at 95°C for 5 sec, annealing step at 58°C for 15sec and extension step at 72°C for 15 sec. Expression of the target mRNA was normalized to the expression of GAPDH mRNA. Experiments were done in triplicates.

### Cytotoxicity assay

To assess possible cytotoxic effects of treatments, lactate dehydrogenase release (LDH Cytotoxicity Detection Kit; TaKara MK401, Shiga, Japan) was measured in the supernatants of HSF cell cultures at 24 h post-treatment. The LDH reaction mix was prepared according to manufacturer’s instructions and kept away from the light. After the addition of the reaction mix, plates were incubated for 30 minutes at room temperature and absorbance was measured at 490nm using a plate reader to determine the percentage of cytotoxicity in each well. Cells lysed with 1% Triton X-100 were taken as positive control and medium without cells as negative control.

### Statistics

Statistical analyses were performed with GraphPad Prism software version 6.01 using one-way ANOVA followed by the Bonferroni’s test for multiple comparisons. Paired t-test was performed for comparison between the absence and presence of drug treatment under the different stimulatory conditions. *p*-values ≤ 0.05 were considered statistically significant. Results are expressed as mean ± standard error “SEM” and as percentage.

## Results

### Immunodetection of several markers of inflammation, osteoclastogenesis and angiogenesis in the synovial tissue of a patient 18 month post-CHIKV infection

**Fig 1:**
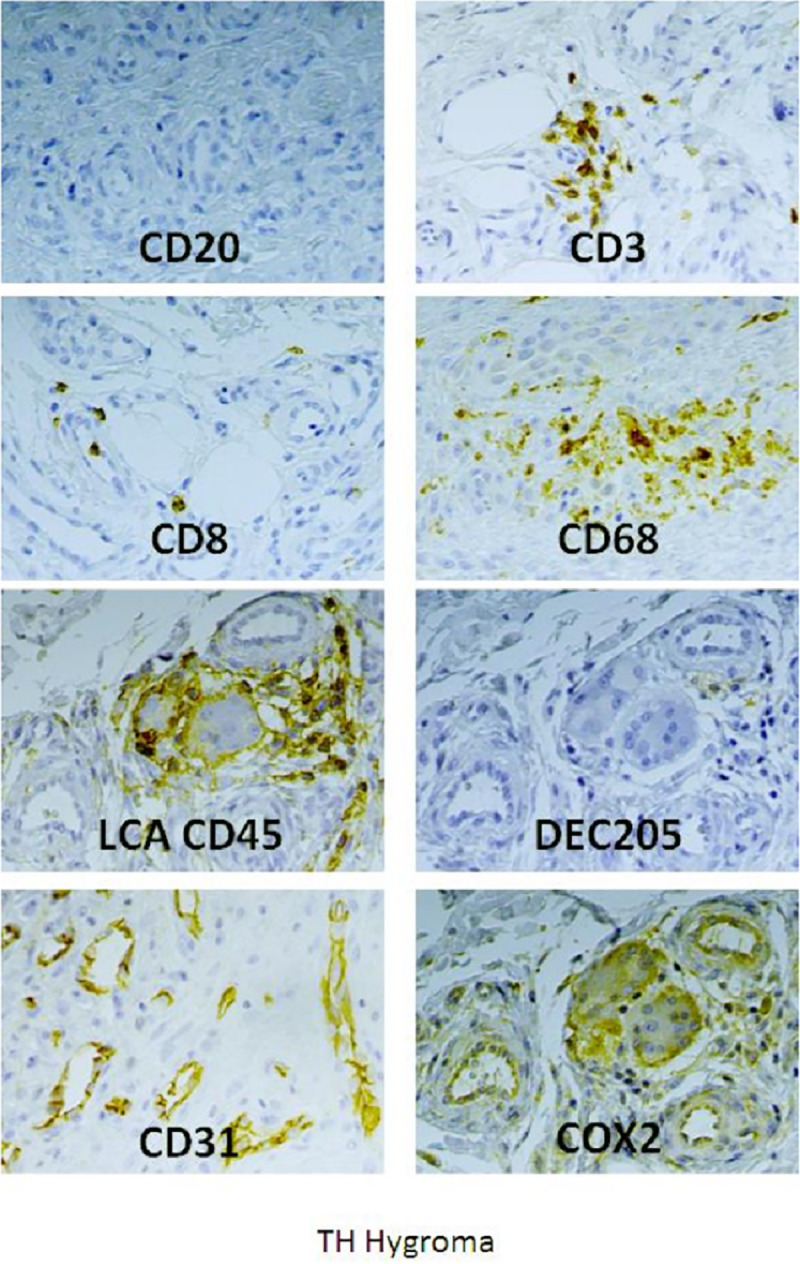
Deciphering the local chronic inflammatory response in the synovial tissue of a patient 18 months post-CHIKV infection. Paraffin wax sections, antigen retrieval and indirect immunoperoxidase stainings were carried out on the biopsy of a patient suffering from chronic arthralgia 18-months post-CHIKV infection. Different markers were tested for the presence of leukocytes (CD45), dendritic cells (DEC205), T cells (CD3 and CD8), macrophages (CD68), angiogenesis (CD31) and prostaglandin biosynthesis (COX-2). Further staining are depicted in S1 Fig, such as for MBP (negative control).

We performed consented histological assessments of a synovial biopsy obtained from a patient suffering from CHIKVD relapses associated with severe chronic arthralgia and requesting surgery to remove his hygroma. We have previously used frozen tissue section of this tissue to show CHIKV persistence with the detection of CHIKV RNA by RTPCR and CHIKV antigens by immunostaining [8]. Follow up experiments were performed on paraffin wax sections and using antigen retrieval (citrate buffer) to address the mechanisms of local inflammation (**Fig 1**). All antibodies were tested on human spleen control tissue sections and validated for immunoreactivities (see example in S1F and S1G Fig). The mouse anti-myelin basic protein (MBP) was used a negative control and displaying background staining (S1A Fig).

Many infiltrating CD45+ leukocytes were present, and subsequent immunostaining indicated that they were mainly CD3+ T cells (including cytotoxic CD8+ cells), whereas no CD20+ /CD79+ B lymphocyte cells were detected (Fig 1). Infiltrating monocytes were identified as CD14+ perivascular cells (S1D Fig) while CD68 staining was localized on ramified, non-amoeboid parenchymal macrophages (Figs 1 and S1 for higher magnification). Very few DEC205+ dendritic cells were also present and around vessels (Fig 1). CD31 and CD34 staining of endothelial cells indicated a process of major angiogenesis which is to be associated with robust detection of VEGF at the level of the extracellular matrix (Figs 1, S1B and S1C). Cell proliferation (KI67 staining) and metalloproteinase expression (MMP-2 staining) were also detected in some areas of the synovial tissue (S1A and S1E Fig). Interestingly, a robust COX-2 staining was detected in the synovial tissue and was associated to vessels, infiltrating leukocytes and multinucleated giant cells (GC) (Fig 1). Further staining indicated that these GC cells were not stained for DEC205 but were strongly expressing CD11c+ marker known to be expressed by macrophage-derived cells. Taking into account the canonical multinucleated features of these GC cells we argued that they could represent a population of intra-synovial osteoclast cells.

**Fig 2:**
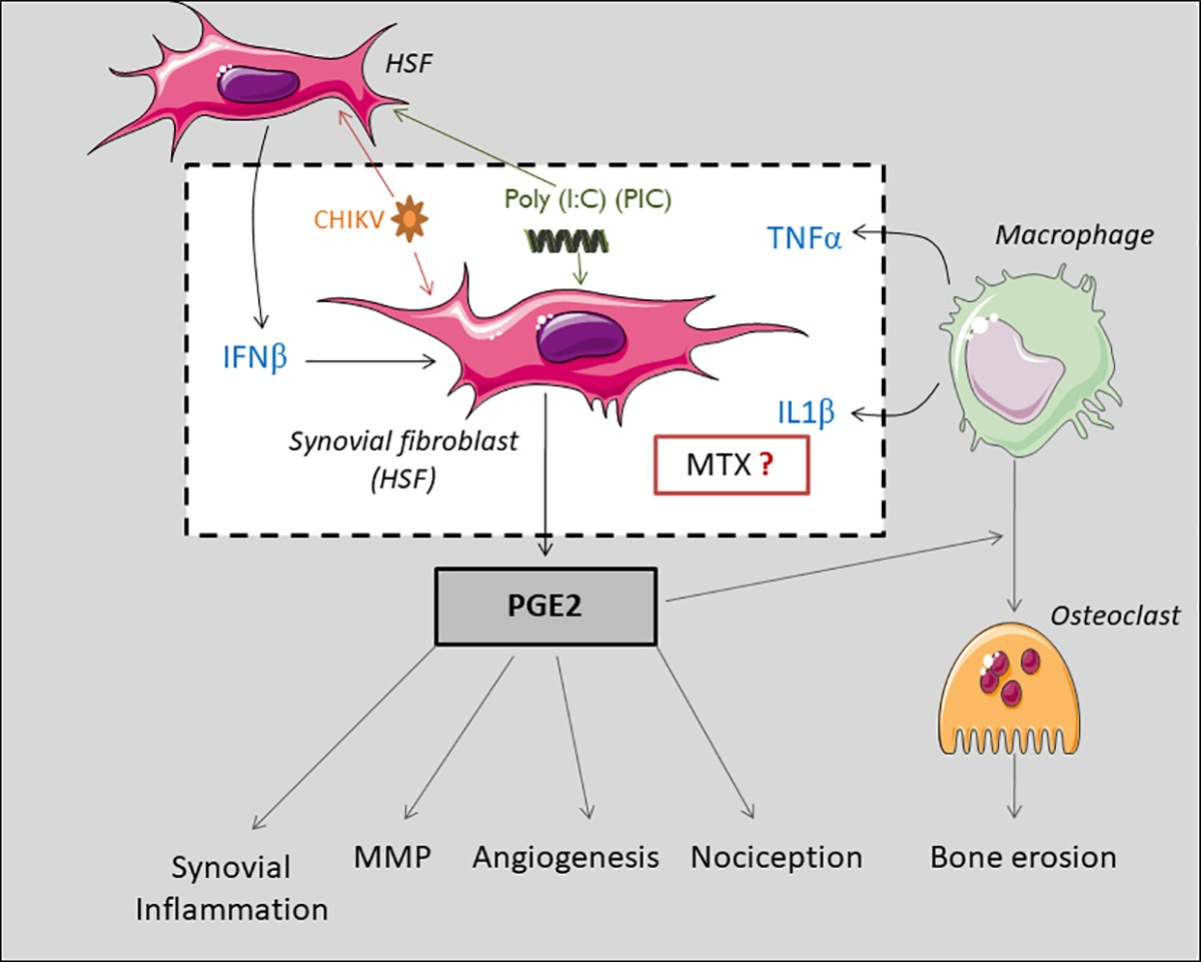
COX-2-mediated prostaglandin response may explain pathogenic features detected in the synovial tissue post-CHIKV infection including metalloproteinase activation, angiogenesis and macrophage infiltration.

All in all, our novel immune-histological assessment argues for a possible COX-2-mediated prostaglandin response in the joint of a patient post-CHIKV infection and which may explain the different pathological features detected in the synovium including metalloproteinase activation, angiogenesis and macrophages infiltration (**Fig 2**). We therefore decided to evaluate *in vitro* the PGE2 biosynthetic pathway and metabolism in response to CHIKV infection and the potential pharmacological effects of MTX in the context of CHIKV persistence in the joints of patients with chronic arthralgia and arthritis (Fig 2).

### Regulated expression of COX-2 involved on PGE2 biosynthesis by a model of primary human synovial fibroblasts

We have assessed by qRT-PCR the mRNA expression of COX-2 and mPGES-1 enzymes of the PGE2 biosynthetic pathway.

We used GAPDH as a housekeeping gene to evaluate the relative expressions of genes of interest.

**Fig 3:**
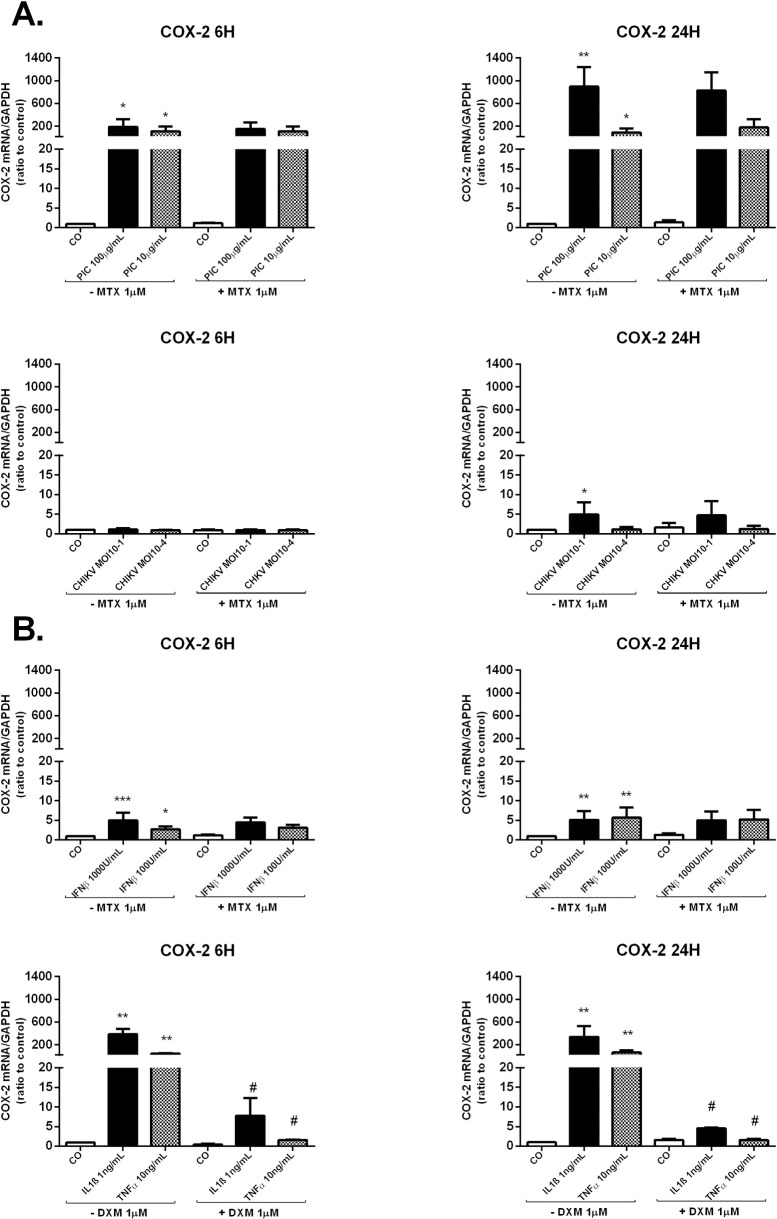
Regulated expression of COX-2 in response to PIC stimulation, CHIKV infection, IFNβ and proinflammatory cytokines (IL-1β and TNFα) in HSF. **A)** COX-2 mRNA levels from HSF infected with CHIKV at MOI 10^−1^ and 10^−4^ or stimulated by PIC 100μg/mL and PIC 10μg/mL in the presence and absence of MTX 1μM for 6h and 24h as assessed by qRT-PCR. **B)** COX-2 gene expression in HSF exposed to IFNβ 1000U/mL and 100U/mL for 6h and 24h and treated or not with MTX 1μM as measured by qRT-PCR. COX-2 mRNA levels were also assessed after IL-1β 1ng/mL and TNFα 10ng/mL stimulation in the presence or not of DXM 1μM treatment. All experiments were done in triplicates. Results are expressed as mean ± standard error and presented as normalized fold increase *vs* control. *: *p*-values ≤ 0.05, **: *p*-values ≤ 0.01 and ***: *p*-values ≤ 0.001 represent significant difference from controls by one-way ANOVA followed by the Bonferroni’s test analysis. #: *p*-values ≤ 0.05, ##: *p*-values ≤ 0.01 signifies statistical difference of samples treated with the drug (MTX or DXM) compared to untreated samples under the different stimulatory conditions by Student’s t-test.

HSF were infected with CHIKV at MOI 10^−1^ and 10^−4^ or stimulated with the viral analog PIC at 100μg/mL or 10μg/mL to screen for direct viral effects on PGE2 biosynthetic pathway. Cells were treated or not with non-toxic concentration of MTX (1μM) (S2 Fig) to evaluate MTX capacity to modulate mRNA expression of the PGE2-synthesizing enzymes (**Fig 3**). Experiments were carried out for 6h to investigate early regulatory mechanisms of MTX. Cellular responses were also evaluated after 24h. HSF are highly permissive to CHIKV. In order to mimic persistence of infectious virus during the chronic phase, CHIKV was used at very low MOIs (10^−1^ and 10^−4^), so that only a small percentage of the cells is infected.

First, in response to PIC 10μg/mL and 100μg/mL stimulations (Fig 3A), the relative expression of COX-2 mRNA was significantly increased at 6h. COX-2 mRNA levels were upregulated up to 120 fold (8.50x10^-1^ ± 5.60x10^-1^, *p*<0.05) post-PIC 10μg/mL stimulation and up to 205 fold (1.47x10^+0^ ± 8.26x10^-1^, *p*<0.05) in response to PIC 100μg/mL stimulation when compared to control cells (7.17x10^-3^± 8.04x10^-4^). At 24h post PIC stimulations, COX-2 mRNA levels were (4.54x10^-1^ ± 2.53x10^-1^, *p*<0.05) post-PIC 10μg/mL (77 fold) and (5.10x10^+0^± 1.35x10^+0^, *p*<0.01) after PIC 100μg/mL (870 fold) when compared to control cells (5.85x10^-3^±1.24x10^-3^).

Second, CHIKV at MOI 10^−1^, but not at the MOI of 10^−4^, significantly upregulated COX-2 mRNA levels at 24h with a fold change of 4 compared to control cells (Fig 3A).

Finally, we found that MTX 1μM co-treatment alone or in the presence of PIC or CHIKV, did not significantly affect the expression of COX-2 mRNA in HSF at 6h and 24h.

PGE2 in the joints of CHIK patients may be induced by direct viral stimulation of the synovial cells. This is to be correlated with previous earlier observations that CHIKV can stimulate PGE2 biosynthesis by cultured fibroblasts via IFN induction [24]. It should also be noted that the proinflammatory cytokines IL-1β and TNFα, known to be produced by recruited activated macrophages, could also strongly stimulate PGE2 production [31]. TNFα and IL-1β were found to induce mPGES-1 and COX-2 expressions [32,33]. In contrast, the synthetic glucocorticoid DXM was shown to exhibit a potent inhibitory effect on PGE2 production by RA synovial fibroblasts [21].

Taken into consideration these previous reports, we were interested in evaluating the capacity of IFNβ to control for the expression of the PGE2-synthesizing enzymes and the potential of MTX to modulate COX-2 and mPGES-1 mRNA levels in response to IFNβ stimulation.

Hence, HSF were exposed to 100U/ml and 1000U/mL IFNβ in the presence or absence of MTX treatment for 6h and 24h (Fig 3B). Cells were also stimulated by IL-1β 1ng/mL and TNFα 10ng/mL and treated or not with DXM 1μM (Fig 3B). DXM was used as a reference drug to ascertain HSF capacity to downregulate PGE2-synthesizing enzymes expressions.

IFNβ at the concentration of 1000U/mL and 100U/ml significantly upregulated COX-2 mRNA levels at 6h and 24h. For example, IFNβ 1000U/mL increased COX-2 relative expression by 5-fold (*p*<0.001) at 6h and by 4-fold (*p*<0.01) at 24h in comparison with unstimulated cells. MTX treatment, used alone or with IFNβ, had not significant effect on COX-2 relative expression.

HSF stimulation by IL-1β 1ng/mL and TNFα 10ng/mL markedly enhanced COX-2 mRNA expression at 6h and 24h. For instance, at 24h post-stimulation, IL-1β 1ng/mL and TNFα 10ng/mL upregulated COX-2 mRNA levels by about 340-fold (*p*<0.01) and 60-fold (*p*<0.01), respectively compared to control cells. DXM significantly inhibited IL-1β and TNFα-induced COX-2 mRNA levels at 6h and 24h.

**Fig 4:**
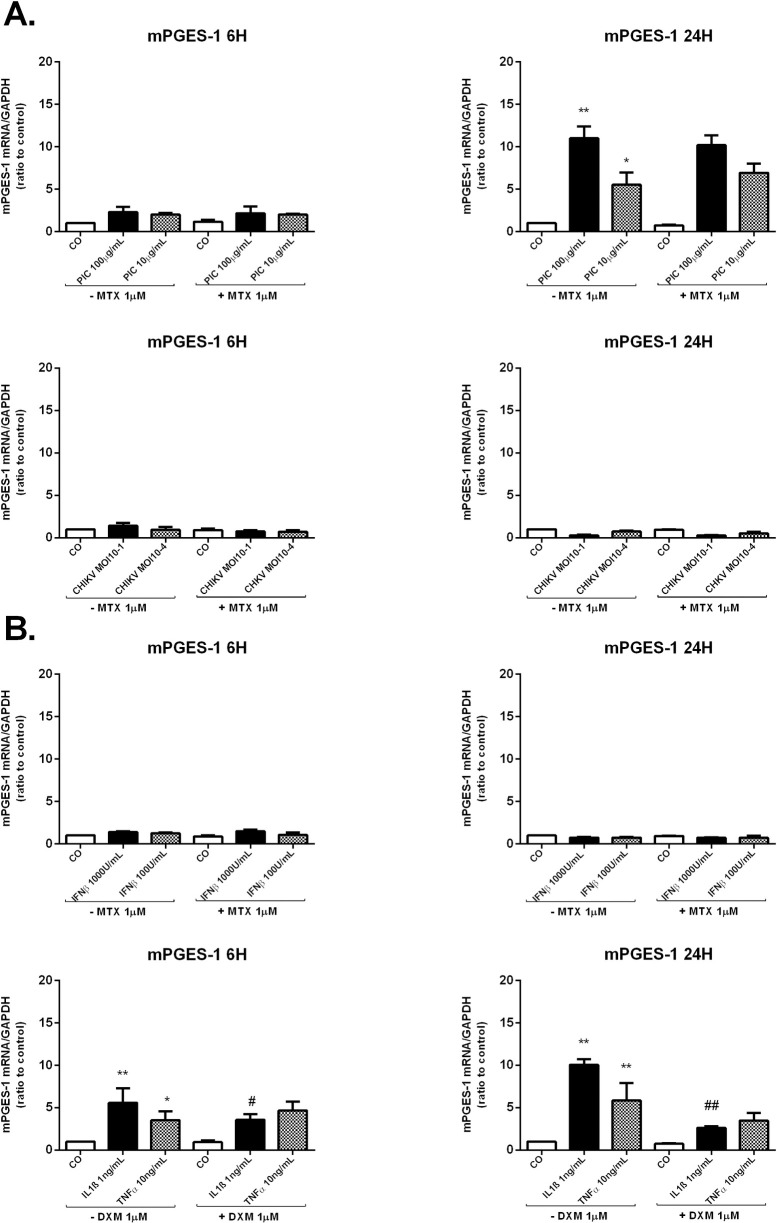
MTX treatment does not interfere with the mRNA expression of mPGES-1 enzyme in HSF infected with CHIKV or exposed to PIC or IFNβ. **A)** mPGES-1 mRNA levels were assessed by qRT-PCR in HSF after CHIKV MOI 10^−1^ and 10^−4^ infection or PIC 100μg/mL and 10μg/mL stimulation in the presence and absence of MTX 1μM for 6h and 24h. **B)** mPGES-1 relative expression from HSF exposed to IFNβ 1000U/mL and 100U/mL +/- MTX 1μM for 6h and 24h as measured by qRT-PCR. mPGES-1 relative expression was also evaluated post-IL-1β 1ng/mL and TNFα 10ng/mL stimulation in the presence or not of DXM 1μM treatment for 6h and 24h. All experiments were done in triplicates. Results are expressed as mean ± standard error and presented as normalized fold increase *vs* control. *: *p*-values ≤ 0.05, **: *p*-values ≤ 0.01 and ***: *p*-values ≤ 0.001 represent significant difference from controls by one-way ANOVA followed by the Bonferroni’s test analysis. #: *p*-values ≤ 0.05, ##: *p*-values ≤ 0.01 signifies statistical difference of samples treated with the drug (MTX or DXM) compared to untreated samples under the different stimulatory conditions by Student’s t-test.

Cellular PGE2 production depends on COX-2 levels, but also on mPGES-1 expression. We assessed by qRT-PCR, mPGES-1 mRNA relative expression in response to CHIKV infection and to viral analog PIC exposure. We have also evaluated MTX capacity to modulate mPGES-1 mRNA expression in the different stimulatory conditions (Fig 4A).

PIC at 10μg/mL and 100μg/mL did not modulate mPGES-1 mRNA levels at 6h. However, mPGES-1 mRNA expressions were significantly upregulated at 24h; (3.18x10^-1^ ± 1.55x10^-1^, *p*<0.05) in response to PIC 10μg/mL stimulation (6 fold increase) and (6.45x 10^−1^ ± 8.62x10^-2^, *p*<0.01) after PIC 100μg/mL stimulation (12 fold increase) when compared to control cells (5.51x 10^−2^± 2.47x10^-2^). The relative expression of mPGES-1 mRNA was not modulated by CHIKV infections at 6h and 24h.

MTX 1μM treatment used alone or in the presence of PIC or CHIKV, did not significantly modulate mPGES-1 mRNA levels in HSF at 6h and 24h.

HSF stimulation by IL-1β 1ng/mL and TNFα 10ng/mL enhanced mPGES-1 mRNA expressions at 6h and 24h (Fig 4B). mPGES-1 mRNA levels were increased by about 10 fold (*p*<0.01) post-IL-1β stimulation and 6 fold (*p*<0.01) post-TNFα stimulation at 24h. DXM did not modulate TNFα-induced mPGES-1 mRNA levels but was effective in inhibiting induced mPGES-1 mRNA levels in response to IL-1β (p<0.01) at 6h and 24h.

IFNβ failed to increase mPGES-1 mRNA levels at 6h and 24h post-stimulation. MTX treatment, used alone or with IFNβ, had not significant effect on mPGES-1 relative expressions.

### MTX treatment effect on the mRNA expression of cPLA2α enzyme involved in AA-release as a substrate for PGE2 synthesis in HSF

Cellular PGE2 production depends on its synthesizing enzymes (COX-2 and mPGES-1) activities as well as on the availability of AA substrate, which is controlled by PLA2 enzymes [17]. cPLA2α was shown to be pivotal for the mobilization of AA and subsequent formation of PGE2. Positioned upstream of the AA-metabolizing enzymes, cPLA2α is considered as the rate-limiting factor for AA release [17].

**Fig 5:**
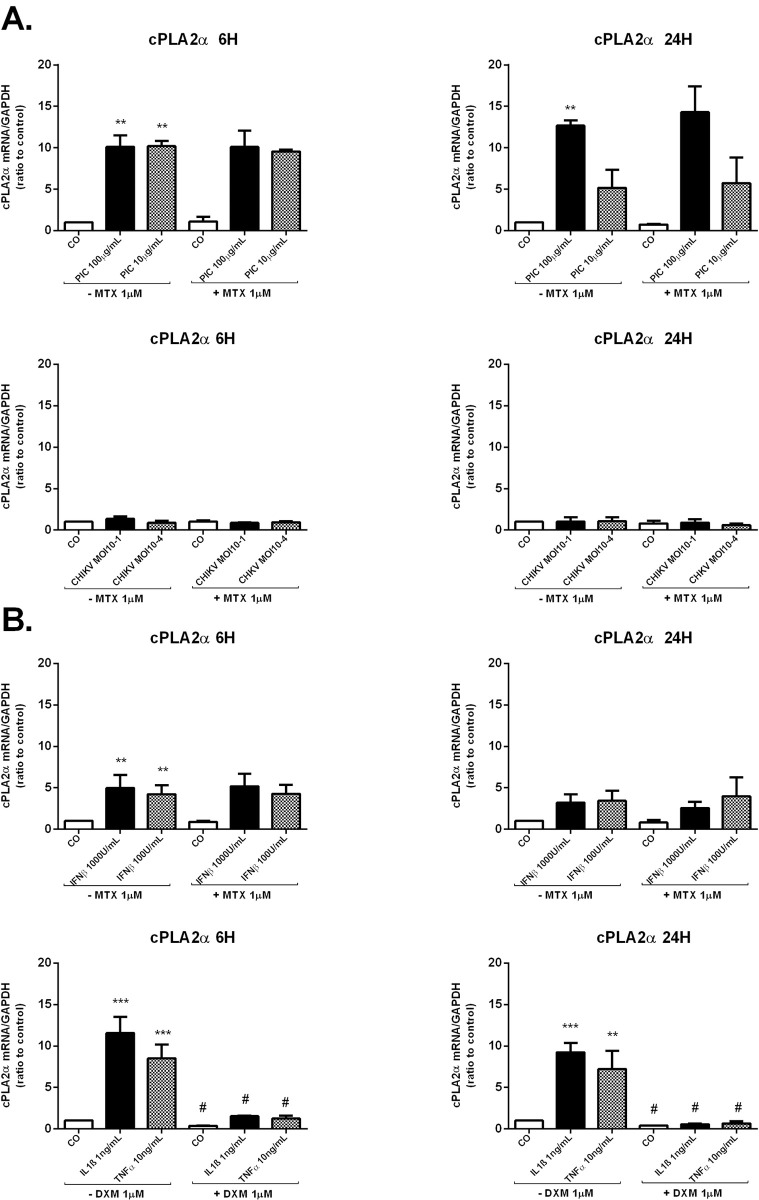
MTX does not modulate mRNA expression of cPLA2 enzyme involved in AA-release for PGE2 synthesis in HSF. **A)** cPLA2 mRNA levels from HSF infected by CHIKV at MOI 10^−1^ and 10^−4^ or stimulated by PIC 100μg/mL and 10μg/mL for 6h and 24h in the absence and presence of MTX 1μM treatment as assessed by qRT-PCR. **B)** mRNA levels of cPLA2 were assessed by qRT-PCR in HSF exposed to IFNβ at the concentration of 1000U/mL and 100U/ml and treated or not with MTX 1μM. HSF were also stimulated by IL-1β 1ng/mL and TNFα 10ng/mL in the absence and presence of DXM 1μM treatment to assess cPLA2 mRNA levels. All experiments were done in triplicates. Results are expressed as mean ± standard error and presented as normalized fold increase *vs* control. *: *p*-values ≤ 0.05, **: *p*-values ≤ 0.01 and ***: *p*-values ≤ 0.001 represent significant difference from controls by one-way ANOVA followed by the Bonferroni’s test analysis. #: *p*-values ≤ 0.05, ##: *p*-values ≤ 0.01 signifies statistical difference of samples treated with the drug (MTX or DXM) compared to untreated samples under the different stimulatory conditions by Student’s t-test.

We wondered whether the CHIKV induced-PGE2 production could be mediated through the induction of AA mobilization by cPLA2α. We therefore decided to evaluate the capacity of CHIKV and the dsRNA PIC to increase the expression of cPLA2α and the potential of MTX to modulate cPLA2α expression. We have investigated by qRT-PCR the relative expression of cPLA2α after either CHIKV infection or PIC stimulation in the presence or absence of MTX (Fig 5A).

The relative expression of cPLA2α was significantly increased in response to PIC 100μg/mL stimulations at 6h and 24h. More than 10-fold increase was observed at 6h (4.45x 10^−1^ ± 1.43x10^-1^, p<0.01) *versus* (4.25x 10^−2^ ± 1.08x10^-2^) and more than 13-fold increase was observed at 24h (3.91x 10^−1^ ± 9.18x10^-2^, p<0.01) *versus* (3.085x 10^−2^ ± 6.36x10^-3^) in cPLA2α mRNA levels after PIC 100μg/mL exposure. In response to PIC 10μg/mL, cPLA2α relative expression was significantly upregulated at 6h (4.31x 10^−1^ ± 1.04x10^-1^, p<0.01) with a fold increase of 10. However, no significant change could be recorded in cPLA2α mRNA levels at 24h after PIC 10μg/mL stimulation. CHIKV infections did not affect cPLA2α relative expression at 6h and 24h. When HSF were treated with MTX alone or combined with CHIKV, no significant difference in cPLA2α mRNA levels was noticed at 6h and 24h. Moreover, MTX did not affect the induction of cPLA2α mRNA levels after PIC treatment.

We next decided to evaluate the capacity of the antiviral agent IFNβ to activate cPLA2α expression in HSF and the potential of MTX to modulate cPLA2α mRNA levels in response to IFNβ exposure. We also aimed to validate the stimulatory effects of the pro-inflammatory cytokines IL-1β and TNFα on cPLA2α expression and to assess DXM inhibitory effects on cPLA2α mRNA levels.

When exposed to IFNβ at the concentration of 1000U/mL and 100U/ml (Fig 4B), HSF significantly increased cPLA2α relative expression at 6h (1.79x 10^−1^ ± 1.42x10^-2^, p<0.01) (5- fold) and (1.58x 10^−1^ ± 3.47x10^-2^, p<0.01) (4-fold), respectively *versus* (3.86x 10^−2^ ± 9.087x10^-3^) in control cells. However, IFNβ 1000U/mL and 100U/ml failed to enhance cPLA2α mRNA levels after a longer incubation time of 24h. MTX treatment did not affect significantly the relative expression of cPLA2α in all tested conditions: alone or together with IFNβ.

Upon stimulation with IL-1β 1ng/mL and TNFα 10ng/mL (Fig 5B), HSF significantly upregulated cPLA2α mRNA expression at 6h and 24h. cPLA2α mRNA levels were increased by about 11-fold post-IL-1β stimulation (*p*<0.001) and 8-fold post-TNFα stimulation (*p*<0.001) at 6h. At 24h, we observed a 9 (*p*<0.001) and 7 (*p*<0.01) fold increases in cPLA2α mRNA levels following IL-1β 1ng/mL and TNFα 10ng/mL stimulation, respectively. DXM significantly suppressed IL-1β and TNFα-induced cPLA2α mRNA to reach basal levels at 6h and 24h.

### MTX effect on the expression of the PGE2 degrading enzyme 15-PGDH by HSF

**Fig 6:**
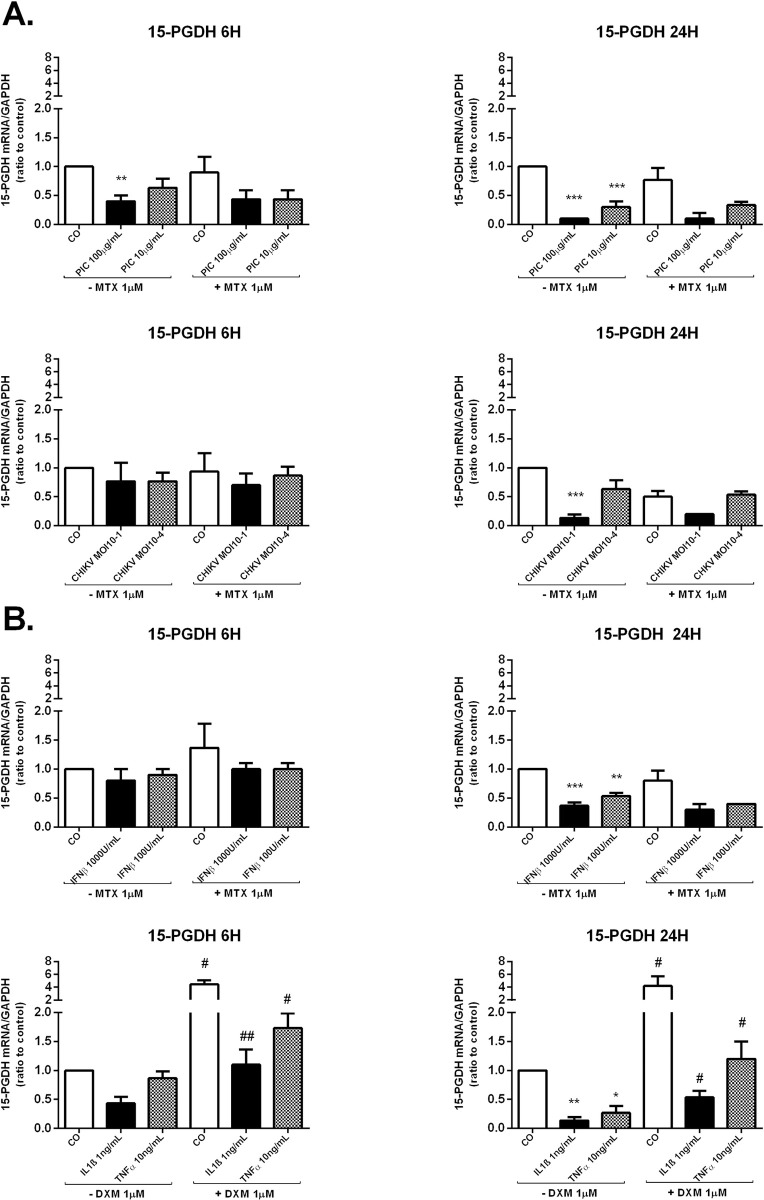
MTX treatment does not affect the expression of the PGE2 degrading enzyme 15-PGDH in HSF. **A)** 15-PGDH mRNA levels from HSF stimulated by PIC 100μg/mL and 10μg/mL or infected by CHIKV at MOI 10^−1^ and 10^−4^ for 6 h and 24 h in the absence and presence of MTX 1μM treatment were evaluated by qRT-PCR. **B)** Relative gene expression of 15-PGDH from HSF after IFNβ 1000U/mL and 100U/mL exposure in the presence and absence of MTX 1μM treatment and post-IL-1β 1ng/mL and TNFα 10ng/mL stimulation in the presence or not of DXM 1μM treatment for 6h and 24h as assessed by qRT-PCR. All experiments were done in triplicates. Results are expressed as mean ± standard error and presented as normalized fold induction *vs* control. *: *p*-values ≤ 0.05, **: *p*-values ≤ 0.01 and ***: *p*-values ≤ 0.001 represent significant difference from controls by one-way ANOVA followed by the Bonferroni’s test analysis. #: *p*-values ≤ 0.05, ##: *p*-values ≤ 0.01 signifies statistical difference of samples treated with the drug (MTX or DXM) compared to untreated samples under the different stimulatory conditions by Student’s t-test.

PGE2 levels are determined by the concerted activities of its synthesizing and its degrading enzymes. Reduced catabolism of PGE2 may contribute to increased levels of PGE2 in the inflamed sites. The key enzyme responsible for metabolic inactivation of prostaglandins, including PGE2_,_ is 15-PGDH [22]. The expression levels of 15-PGDH in human synovium were found to be reduced in RA synovium compared to normal synovium [34]. We aimed to investigate the capacity of CHIKV and MTX to modulate the expression of the PGE2-degrading enzyme 15-PGDH in HSF. mRNA levels of 15-PGDH were assessed by qRT-PCR at 6h and 24h after CHIKV or PIC exposure in the presence or absence of MTX 1μM treatment (Fig 6A).

Exposure to PIC 100μg/mL significantly decreased 15-PGDH gene expression in HSF at 6h and 24h (Fig 4A). 15-PGDH mRNA levels were (2.69x 10^−4^ ± 8.64x10^-5^,*p* ≤ 0.01) (3 fold decease) at 6h and (6.64x 10^−5^ ± 1.25x10^-5^, *p*≤ 0.001) (10-fold decease) at 24h when compared to control cells (6h, 6.77x 10^−4^ ± 1.26x10^-4^); (24h, 6.73x 10^−4^ ± 5.89x10^-5^). After 6h of incubation with PIC 10μg/mL, 15-PGDH mRNA levels were similar to control values. However, PIC 10μg/mL significantly reduced by 3-fold 15-PGDH mRNA levels (*p*<0.001) at 24h post-stimulation.

CHIKV infections did not significantly affect the expression of 15-PGDH mRNA at 6h whereas CHIKV at the MOI of 10^−1^ significantly decreased by 7-fold (*p*<0.001) 15-PGDH mRNA levels at 24h post-infection (6.40x 10^−5^± 2.21x10^-5^) *versus* (4.71x 10^−4^± 5.73x10^-5^) in mock-infected control cells. No significant difference in 15-PGDH mRNA levels was observed after MTX treatment alone or combined with PIC or CHIKV.

We next evaluated 15-PGDH relative expression in HSF after IFNβ 1000U/mL and 100U/mL exposure in the presence or not of MTX 1μM. We also investigated the effects of individual and combined treatment with IL-1β 1ng/mL, TNFα 10ng/mL and DXM 1μM on 15-PGDH relative expression (Fig 6B). IFNβ treatment did not significantly change HSF expression levels of 15-PGDH at 6h. In contrast, 15-PGDH gene expression was significantly decreased at 24h by about 3-fold (*p*<0.001) when incubated with IFNβ 1000U/mL and by about 2-fold (*p*<0.01) after IFNβ 100U/mL exposure. MTX treatment had no significant effect on 15-PGDH relative expression after IFNβ stimulation at 6h and 24h.

IL-1β 1ng/mL and TNFα 10ng/mL significantly suppressed 15-PGDH mRNA expression at 24h by about 11-fold (*p*<0.01) and 4-fold (*p*<0.05), respectively compared to control cells. DXM used alone significantly upregulated 15-PGDH mRNA levels in control cells at 6h and 24h. More importantly, DXM significantly decreased IL-1β 1ng/mL and TNFα 10ng/mL inhibitory effects on 15-PGDH mRNA levels at 6h and 24h.

## Discussion

The physiopathological mechanism of chronic CHIK arthritis and arthralgia is still incompletely understood and may be mediated at least in part by elevated PGE2 production in the synovial fluid of chronically infected patients. Activated synovial tissue cells, recruited leukocytes and endothelial cells could contribute to PGE2 release into the synovial fluid and tissue [21]. We made the hypothesis that PGE2 in the joints of CHIK patients may be induced by direct viral activation (CHIK viral particles or persistent CHIKV RNA) in the synovial cells or regulated indirectly through induced interferon production or increased inflammatory cytokines secretion (such as IL-1β and TNFα) by recruited macrophages. We argued that PIC could be used to mimic the effect of viral dsRNA present in the joint during the chronic phase of CHIKV [8,30]. Hence, we investigated potential CHIKV direct and indirect effects on HSF PGE2 biosynthetic pathway.

Our histological assessments were restricted to a synovial biopsy collected from one patient with CHIKVD relapses. However, our novel immunohistological data strongly support the aforementioned hypothesis for several reasons. First, we observed a strong leukocyte infiltration (CD45 staining) and synovial cell proliferation (Ki67 staining) which may argue for a status of cell activation favoring the production of cytokines by both cell types. Growth factors such as VEGF detected in the synovial tissues may also contribute to angiogenesis (as indicated by robust CD31 and CD34 staining) and further contributing possibly to leukocyte infiltration. T cells (CD3 and CD8 + cells) may produce cytokines such as IFN-gamma and contributing to the activation of CD14+ monocytes and CD68+ macrophages. Interestingly, B cells (CD20 and CD79a staining) were absent and this is to be correlated to the scarce detection of dendritic cells (Dec205+) in the synovial tissue in sharp control to spleen control tissue sections. This latter point is not in favor of an autoimmune adaptive immune response driving for instance the production of anti-citrullinated antibodies. More importantly, our data also revealed the presence of a strong expression of COX-2 notably by multinucleated GC and which were also identified as CD11c+ macrophage cells and possibly corresponding to intra-synovial osteoclasts. Although a robust COX-2 expression was detected, we were not able to compare COX-2 staining in non-diseased synovial tissue. However, previous studies reported the absence of COX-2 detection in normal synovial membranes [35,36]. Not all makers of inflammation were detected and we noted the absence of NOS staining.

Next, we wanted to analyze the molecular mechanisms which may control COX-2 and more globally prostaglandin expression in the joint of a patient with chronic arthralgia and arthritis. To this aim, we used our well-validated model of primary synovial fibroblast. We first analyzed the expression of the PGE2 synthesizing enzymes COX-2 and mPGES-1 in HSF infected with CHIKV or stimulated with the viral analog PIC to screen for direct viral effects on PGE2 synthesis. There is considerable evidence that low dose MTX is an effective agent for the treatment of chronic CHIKV arthritis. The underlying mechanism of action is poorly understood. We therefore assessed MTX capacity to modulate the expression of several enzymes of the PGE2 biosynthetic pathway and metabolism.

Earlier studies have shown that intra-articular injection of PIC caused acute synovitis in rats with increased concentration of PGE2 in the synovial tissue [37]. Furthermore, PIC at 10–100μg/mL was shown to induce PGE2 production in cultured synovial fibroblasts [25,38,39]. Our data showed that the transcription levels of COX-2 and mPGES-1 were strongly upregulated in response to PIC 100μg/mL and 10μg/mL stimulation in HSF.

In contrast to our results, Gutierrez-Venegas and Rodriguez-Perez [40] demonstrated that PIC reduced COX-2 expression and PGE2 production induced by histamine in human gingival fibroblasts. The differences observed in the two studies are possibly due to the different cell types and stimulatory agents used. However, another study has shown that, in Mesenchymal stem cells (MSCs), PIC 10μg/mL increased the mRNA relative expression of COX-2 at 12h and 24h post-stimulation [41]. In another study, PIC was shown to increase the expression of mPGES-1 and COX-2 in rat primary microglia [42].

There is a paucity of studies assessing CHIKV effects on PGE2 biosynthetic pathway. Fitzpatrick et al. reported that PGE2 is highly expressed by CHIKV-infected human fibroblasts [24]. Here, we demonstrated that CHIKV at MOI 10^−1^ significantly upregulated COX-2 mRNA levels up to 4-fold at 24h post-infection. However, mPGES-1 relative expression was not modulated by CHIKV infection. Thus, CHIKV infection may induce PGE2 biosynthesis through the induction of COX-2 expression in HSF. In accordance with our data, the CHIKV T-SBY strain isolate was shown to increase COX-2 expression in the human embryonic kidney epithelial cell line 293T [43].

MTX, used alone or combined with PIC or with CHIKV, did not affect the expression of COX-2 and mPGES-1 mRNA in HSF at 6h and 24h.

An anti-inflammatory effect of MTX has been suggested by its rapid onset of action [44] but its effect on PGE2 synthesis remains controversial. Administered in a rabbit model of antigen induced arthritis, MTX was found to decrease the intra-articular production of PEG2 compared to saline treated controls [45]. Added *in vitro*, MTX caused a dose dependent decrease on IL-1 induced PGE2 production by RA synovial cells, without affecting COX-2 mRNA expression [46]. Other studies pointed to no effect of MTX on PGE2 synthesis. MTX did not modulate mPGES-1 and COX-2 expressions in RA synovial tissue biopsies [47]. Moreover, MTX had no effect on IL1-induced PGE2 release by RA synovial fibroblasts [48].

We next studied the effect of IFNβ stimulation on the expression of the PGE2-synthesizing enzymes COX-2 and mPGES-1. We found that IFNβ at the concentration of 1000 and 100U/mL significantly upregulated COX-2, but not mPGES-1, mRNA levels in HSF at 6h and 24h.

These results corroborate former published studies; Yaron et al. showed that IFN may stimulate PG production in a dose dependent manner by synovial fibroblasts [25]. Furthermore, IFN produced by CHIKV-infected foreskin fibroblasts was suggested to induce PGE2 release [24].

We also assessed the effect of CHIKV infection and PIC stimulation on cPLA2α mRNA expression in HSF and the potential of MTX to modulate cPLA2α expression.

Stimulation of cultured HSF with PIC significantly upregulated cPLA2α mRNA levels at 6h and 24h. In agreement with our findings, Ruipérez et al. showed that activation of the P388D1 and RAW2647.1 macrophage-like cells with the dsRNA viral analog PIC resulted in AA mobilization through the stimulation of cPLA2 enzyme [49]. Pindado et al. found that PIC treatment of RAW264.7 murine macrophage-like cells promoted cPLA_2_α-mediated AA release and COX-2-mediated PGE2 production. Furthermore, inhibition of cPLA_2_α by selective pharmacological strategies dramatically reduced AA release in response to PIC [50].

CHIKV infection failed to modulate cPLA2α mRNA expression in HSF at 6h and 24h.

This result is in concordance with that of Chalaem et al. who also showed that CHIKV did not affect PLA2 transcription levels in the human embryonic kidney epithelial cell line 293T [43]. Unlike CHIKV, PIC was able to strongly upregulate all enzymes of the PGE2 biosynthetic pathway. PIC is a synthetic analogue of viral dsRNA, whereas CHIK viral particles are composed of a single-stranded RNA genome and proteins. Viral proteins can interact with host patterns and interfere with host antiviral response [51,52]. One interesting observation reported by Fros and colleagues is that CHIKV Nonstructural Protein 2 (nsP2) can inhibit type I/II interferon-stimulated JAK-STAT signaling [52]. The nsP2 protein has been also found to be able to induce a shutdown of host transcription [53]. nsP2-induced transcriptional shut off can explain the inability of CHIKV-infected cells to strongly upregulate enzymes of the PGE2 biosynthetic pathway, as seen after PIC stimulation.

We found that MTX treatment had no effect on cPLA2α mRNA levels in basal conditions as well as after PIC stimulation and CHIKV infection.

In a cohort of 100 patients, Michaels et al. showed that MTX treatment did not affect the increased serum PLA2 activity in patients with RA [18]. In a mouse model of collagen induced arthritis (CIA), MTX was used as a reference drug to compare its effects with those of two cPLA2α inhibitors (AVX001 and AVX002). MTX, as well as cPLA2α inhibitors, were found to significantly reduce elevated plasma PGE2 levels in CIA mice. However, MTX capacity to decrease cPLA2α expression was not assessed in this study [19].

We found that IL-1β 1ng/mL and TNFα 10ng/mL upregulated cPLA2α mRNA expression in HSF and DXM significantly suppressed IL-1β and TNFα-induced cPLA2α mRNA levels. It has been previously shown that TNFα and IL-1β activate PLA2 enzymes [54]. IL1β was found to induce mRNA and protein levels of cPLA2 in RA synovial fibroblasts contributing to increased production of PGE2 [55]. mRNA expression of cPLA2 were shown to be induced by IL-1β and TNFα in gingival fibroblasts. The anti-inflammatory steroid DXM was able to block the induction of cPLA2 mRNA by IL-1β and TNFα [56].

15-PGDH is the key enzyme responsible for the metabolic inactivation of PGE2 and associated eicosanoids. 15-PGDH expression levels were found to be reduced in RA synovium compared to normal synovium [34].

We demonstrated that PIC stimulation as well as CHIKV MOI 10^−1^ infection significantly reduced 15-PGDH mRNA levels in HSF. MTX treatment did not interfere with the expression of 15-PGDH in basal conditions as well as after PIC stimulation or CHIKV infection.

15-PGDH was shown in recent published microarray data [57] to be strongly downregulated by the dsRNA PIC in cultured normal human epidermal keratinocytes (NHEKs). This downregulation of 15-PGDH by PIC, together with an observed marked upregulation of COX-2, is consistent with our observations about the ability of the dsRNA PIC to modulate the expression of several enzymes of the PGE2 biosynthetic pathway.

MTX treatment ability to affect the expression levels of 15-PGDH was assessed in both RA biopsies *ex vivo* and in cultured RAFLSs. No effects of MTX on synovial 15-PGDH expression were detected [47]. However, Kim et al. reported an increase in 15-PGDH expression levels in RAFLS after MTX 10 nM treatment [34].

We demonstrated that IFNβ 1000U/mL and 100U/mL significantly decreased 15-PGDH mRNA expression in HSF at 24h. MTX treatment did not modulate IFNβ-inhibitory effect on 15-PGDH mRNA levels. We also found that IL-1β 1ng/mL and TNFα 10ng/mL suppressed 15-PGDH mRNA expression at 24h. DXM significantly reduced the inhibitory effects of IL-1β 1ng/mL and TNFα 10ng/mL on 15-PGDH mRNA levels.

Consistent with our observations, treatment of primary cultures of trophoblast cells with IL-1β or TNFα resulted in decreased 15-PGDH mRNA expression [58]. IL-1β and TNFα were shown to downregulate 15-PGDH expression in A549 human lung adenocarcinoma cells with concomitant induced COX-2 expression [59]. DXM and other glucocorticoids were found to induce 15-PGDH expression in a time- and dose-dependent manner in A549 cells [60]. DXM may, in part, exert its anti-inflammatory actions by enhancing the catabolism of PGE2. Paradoxically, Gheorghe et al. reported a trend toward reduced 15-PGDH expression in RA patients synovium after intra-articular glucocorticoid therapy [47].

**Fig 7:**
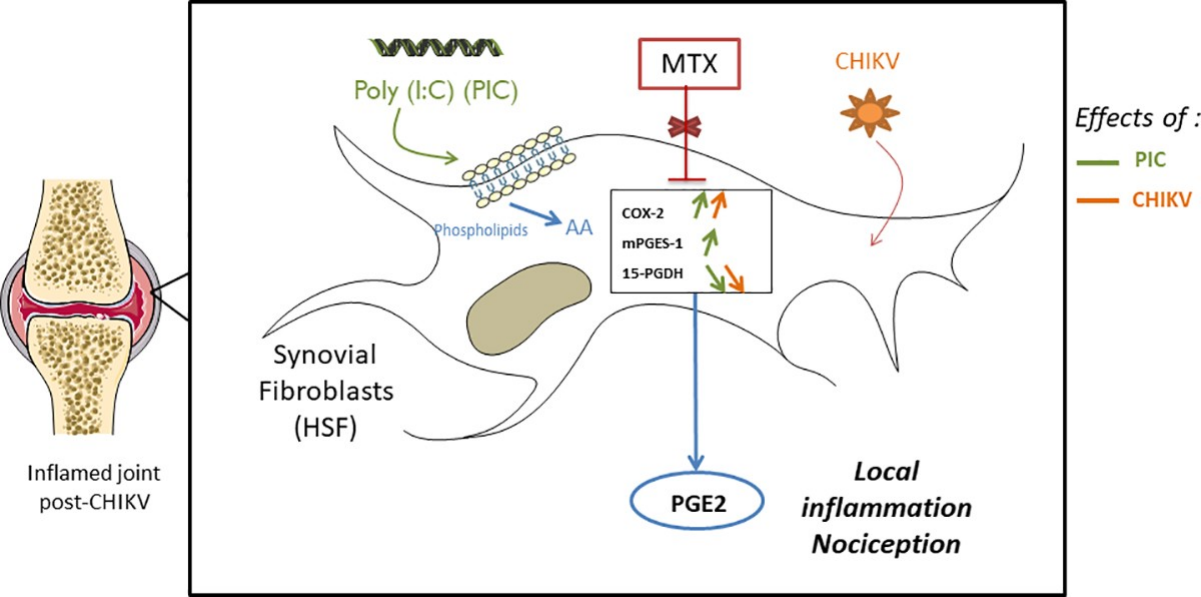
Effect of methotrexate (MTX) treatment on the mRNA expression of major enzymes involved in PGE2 metabolism and in the context of CHIKV infection linked to chronic arthritis. (Arachidonic acid, AA), Prostaglandin E2 (PGE2).

In conclusion, we demonstrated that HSF can strongly up regulate all major enzymes involved in the PGE2 biosynthetic pathway in response to viral RNA (PIC). In contrast, only COX-2 expression was upregulated by CHIKV-infected HSF. The major PGE2 degrading enzyme (15-PGDH) was downregulated in response to CHIKV infection. IFNβ was able to modulate the expression of almost all enzymes of PGE2 synthesis and metabolism. PGE2 levels in the joint of CHIK patients may therefore be regulated by CHIKV-mediated IFNβ production or by proinflammatory cytokines such as IL-1β and TNFα produced by recruited macrophages. MTX treatment did not interfere with the expression of cPLA2α, COX-2, mPGES-1 or 15-PGDH in all tested conditions (Fig 7). The mechanisms involved in the therapeutic activity of MTX to explain the control of arthralgia and arthritis post-CHIKV chronic infection seems to be mediated independently of the PGE2 regulated response and remains to be ascertained.

## Supporting information

S1 Fig Immunostaining of synovial tissue of a patient (hygroma) 18 months post chikungunya. Immunoperoxidase staining of paraffin wax tissue sections (DAB brown revelation) and hematoxylin counterstaining in blue of the nuclei were carried out on a biopsy of a patient suffering from CHIKD replases 18-months post-infection. Different markers were tested to assess cell proliferation (KI67; S1A Fig), immune cell invasion (CD45; S1B Fig), presence of perivascular monocytes (CD14; S1D Fig) and parenchymal macrophages (CD68; S1D Fig), angiogenesis (CD34 and VEGF; S1B and S1C Fig), synthesis of Nitric Oxide by NOS (S1C Fig), metalloprotease (MMP2; S1E upp Fig) and prostaglandin biosynthesis (COX-2; S1E Fig). Some antibodies (anti-DEC205 and anti-CD11c; S1F and S1G Fig) were checked for reactivity using spleen tissue sections.
(PDF)

S2 Fig MTX and DXM at 1 μM do not have cytotoxic effects on HSF. **A)** HSF were incubated with PIC 100μg/mL, CHIKV MOI 10–1, IFNβ 1000U/mL, IL1-β 1ng/mL and TNFα 10ng/mL in the presence or not of MTX 1μM or DXM 1μM for 24h. Cytotoxicity was monitored by measuring percentage of LDH released in culture supernatants. Results are from 3 independent experiments. ***p < 0.01 *vs* the control, *p < 0.05 *vs* the control, by one-way ANOVA followed by the Bonferroni’s test.
(TIFF)
