# Supplementary material for: Robust COX-2-mediated prostaglandin response may drive arthralgia and bone destruction in patients with chronic inflammation post-chikungunya
Source: PLoS Negl Trop Dis. 2021 Feb 17;15(2):e0009115. doi: 10.1371/journal.pntd.0009115 (PMC7920362; doi:10.1371/journal.pntd.0009115)
Supplement: S1 Fig — Immunoperoxidase staining of paraffin wax tissue sections (DAB brown revelation) and hematoxylin counterstaining in blue of the nuclei were carried out on a biopsy of a patient suffering from CHIKD replases 18-months post-infection. Different markers were tested to assess cell proliferation (KI67; S1A Fig), immune cell invasion (CD45; S1B Fig), presence of perivascular monocytes (CD14; S1D Fig) and parenchymal macrophages (CD68; S1D Fig), angiogenesis (CD34 and VEGF; S1B and S1C Fig), synthesis of Nitric Oxide by NOS (S1C Fig), metalloprotease (MMP2; S1E upp Fig) and prostaglandin biosynthesis (COX-2; S1E Fig). Some antibodies (anti-DEC205 and anti-CD11c; S1F and S1G Fig) were checked for reactivity using spleen tissue sections. (PDF) [file pntd.0009115.s001.pdf]

Supplemental Figure 1A

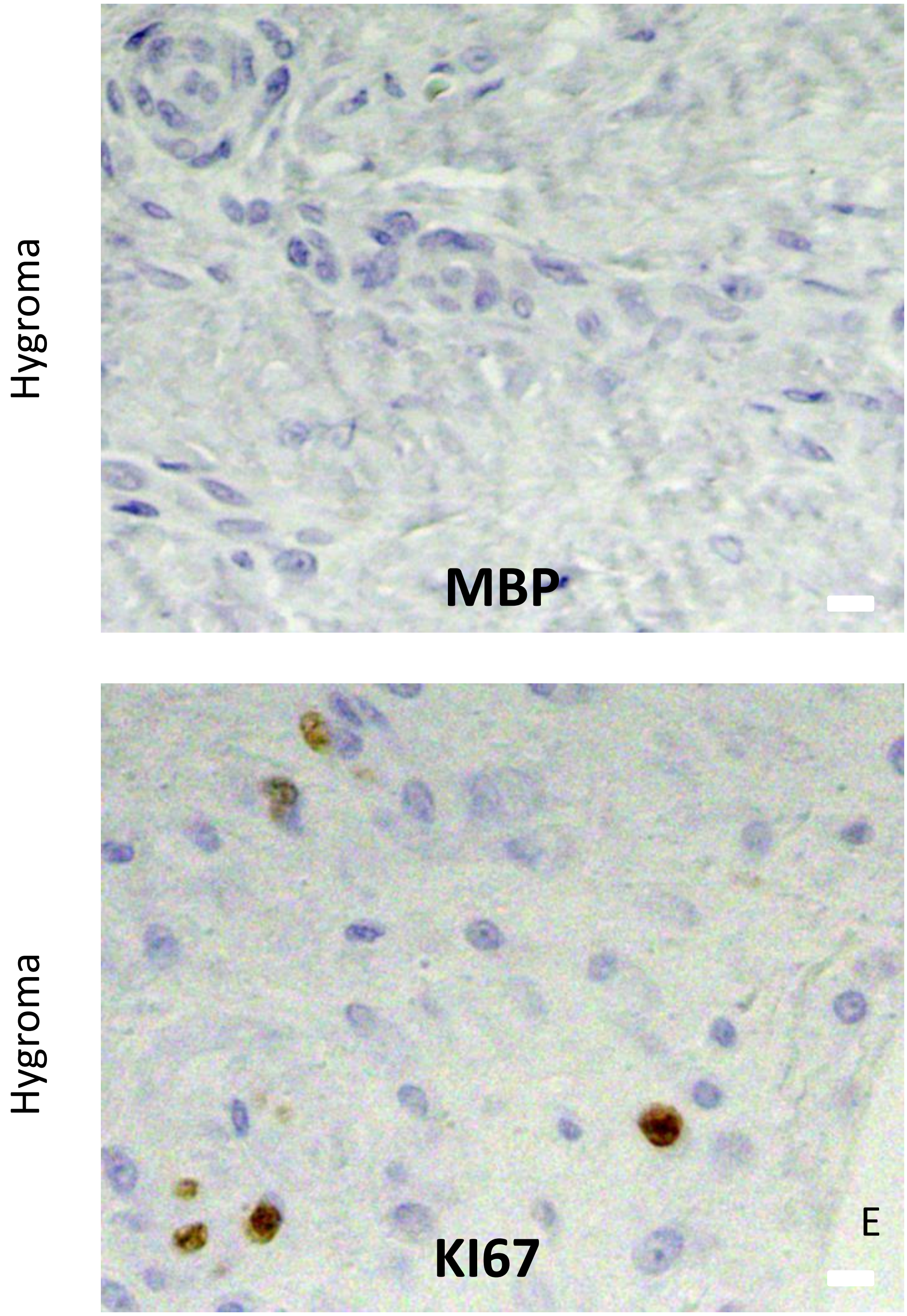

Negative control (MBP)
Cell proliferation (KI67)

Supplemental Figure 1B

Hygroma

Hygroma

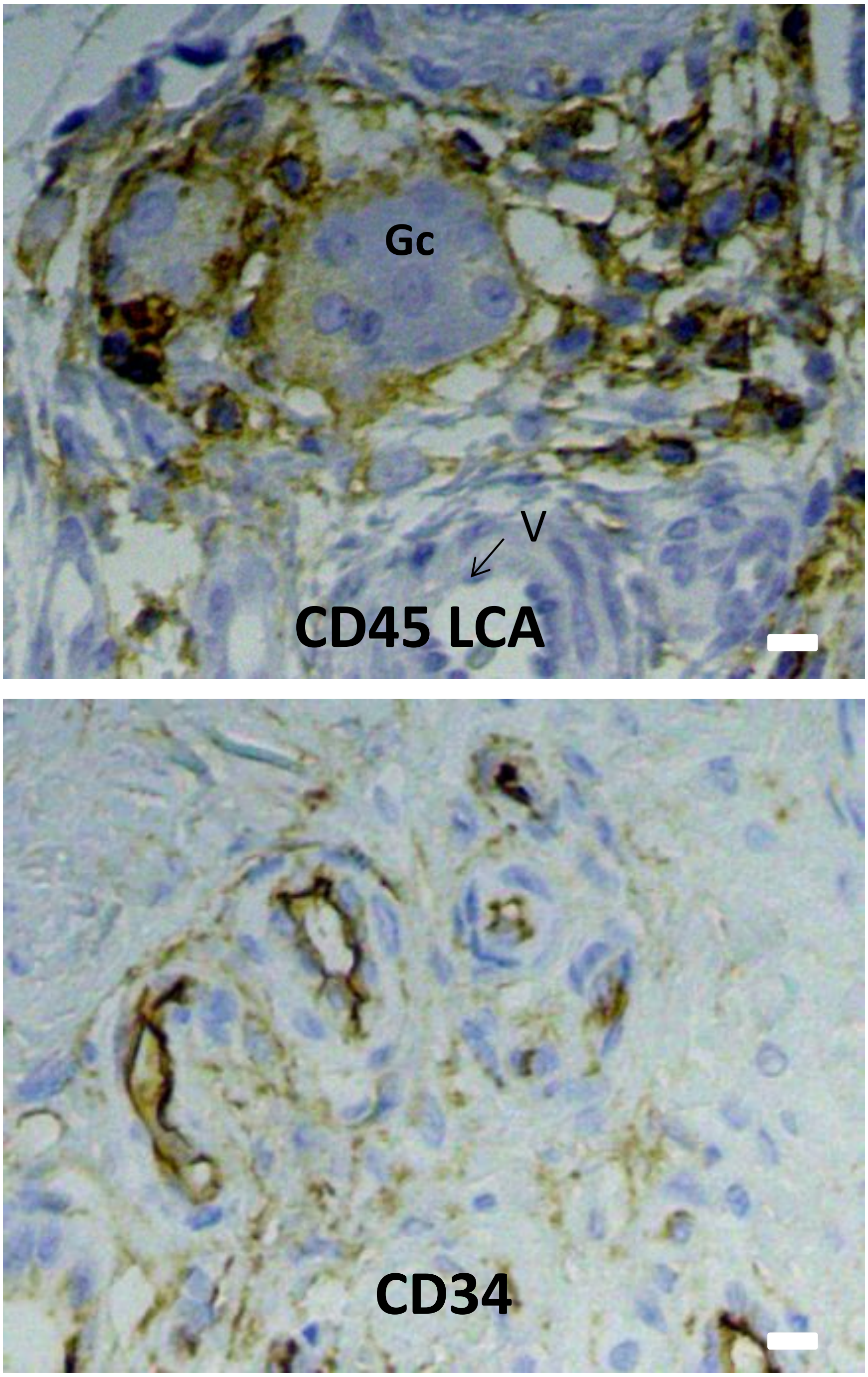

Immune cell invasion (CD45)
Major angiogenesis (CD34)

Supplemental Figure 1C

Hygroma

Hygroma

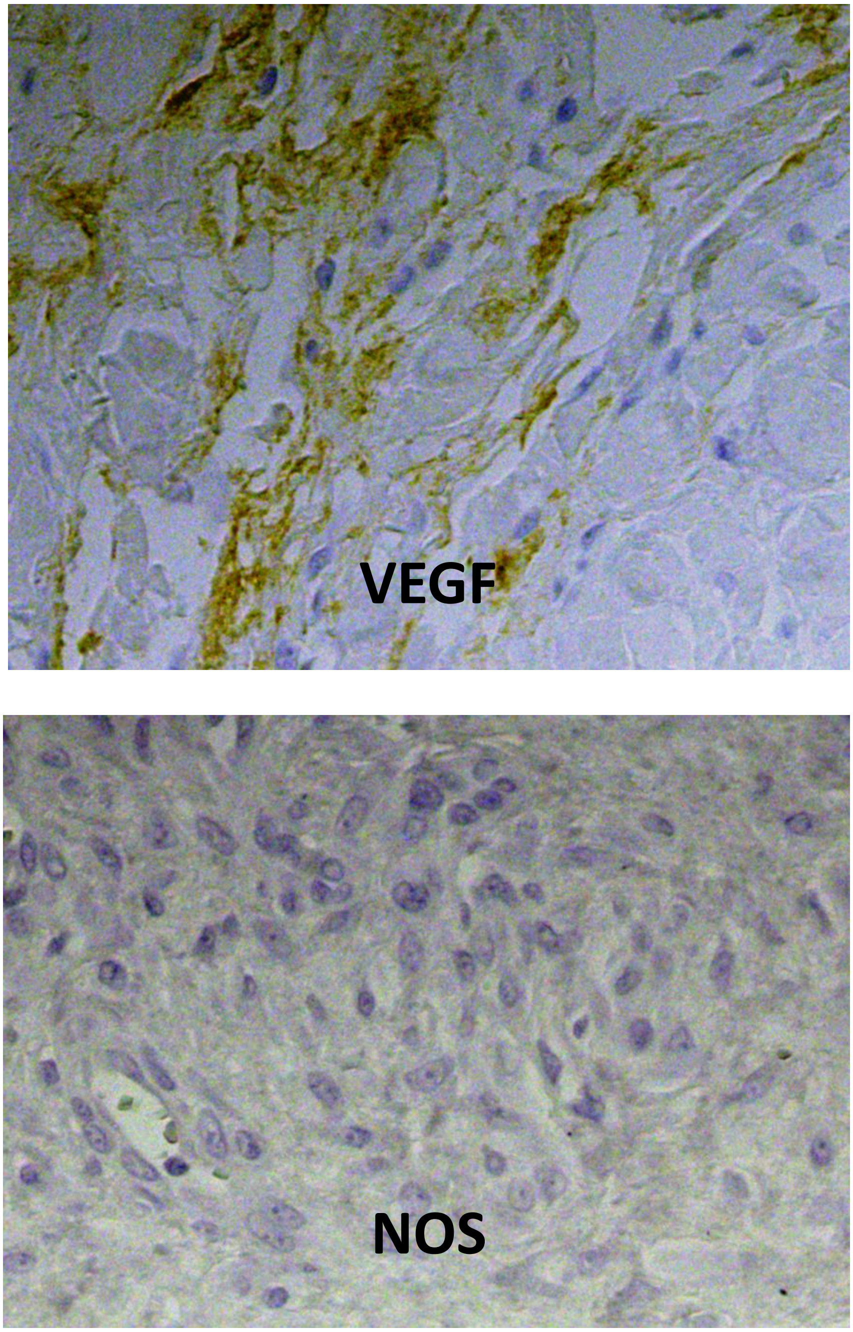

Proangiogenic factor, VEGF
Synthesis of Nitric Oxide by NOS

Supplemental Figure 1D

Hygroma

Hygroma

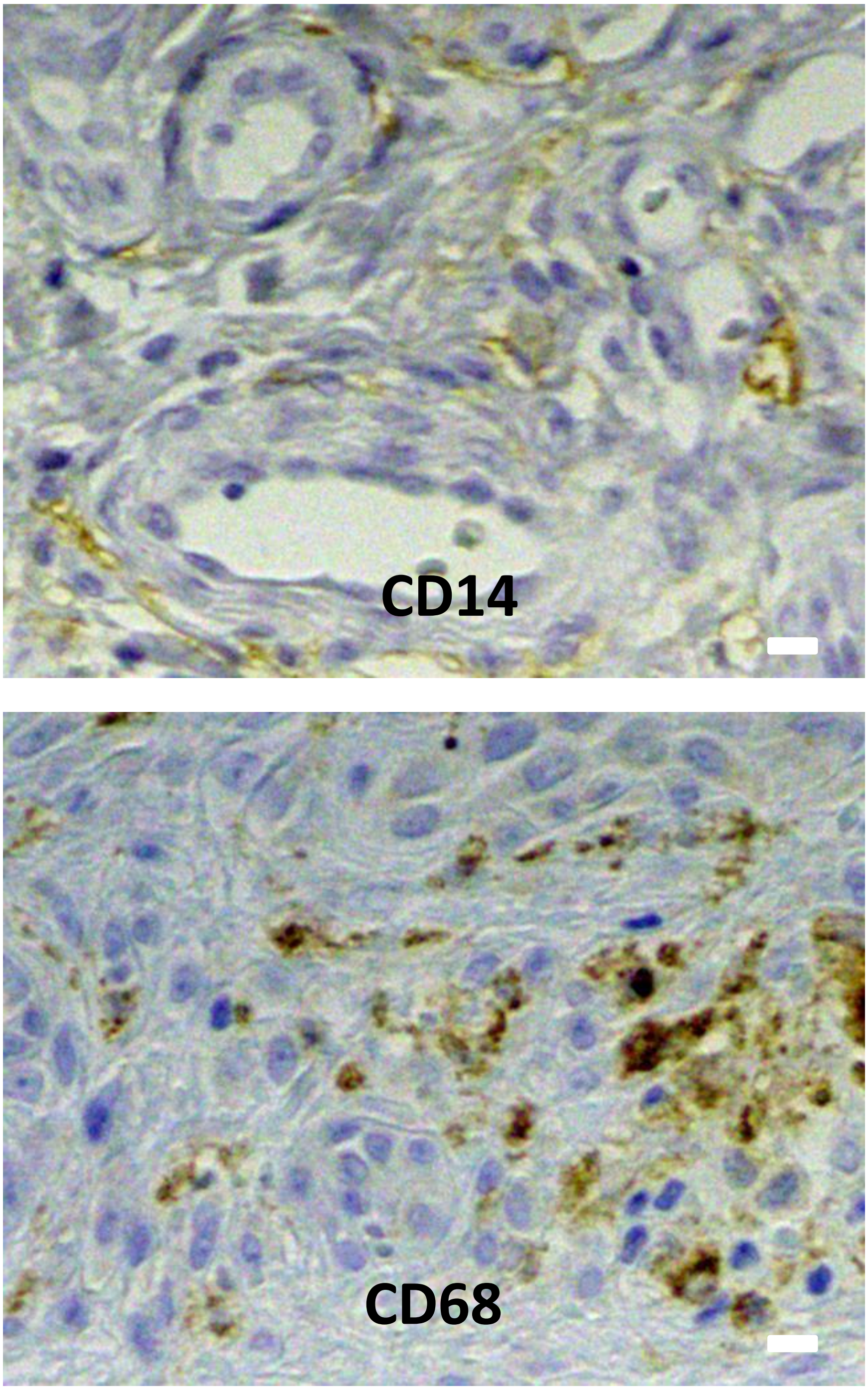

Monocyte (CD14, perivascular)
Parenchymal MØ cells (CD68)

Supplemental Figure 1E

Hygroma

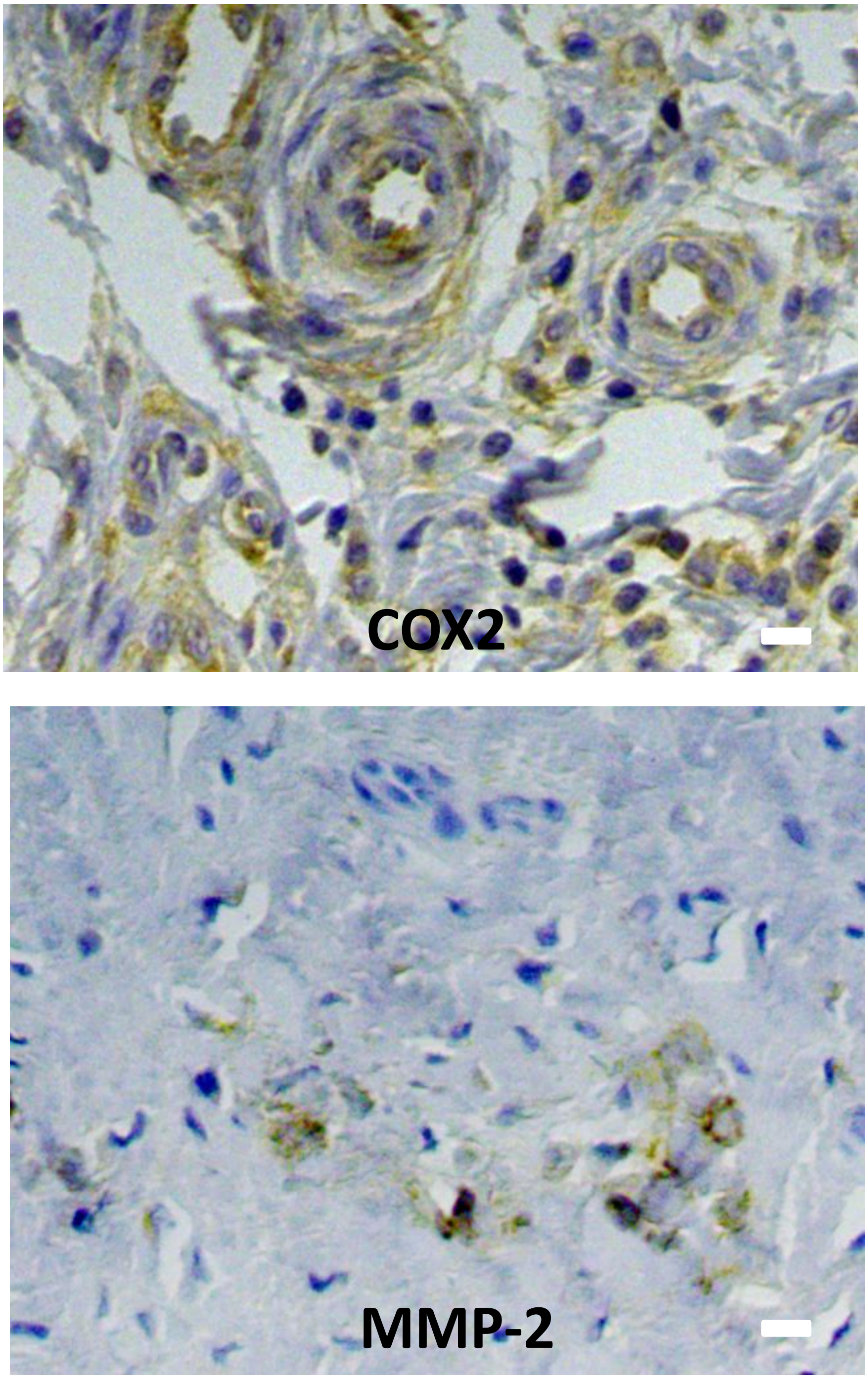

Hygroma

Perivascular COX2
Metalloprotease MMP2

Supplemental Figure 1F

Hygroma

Spleen

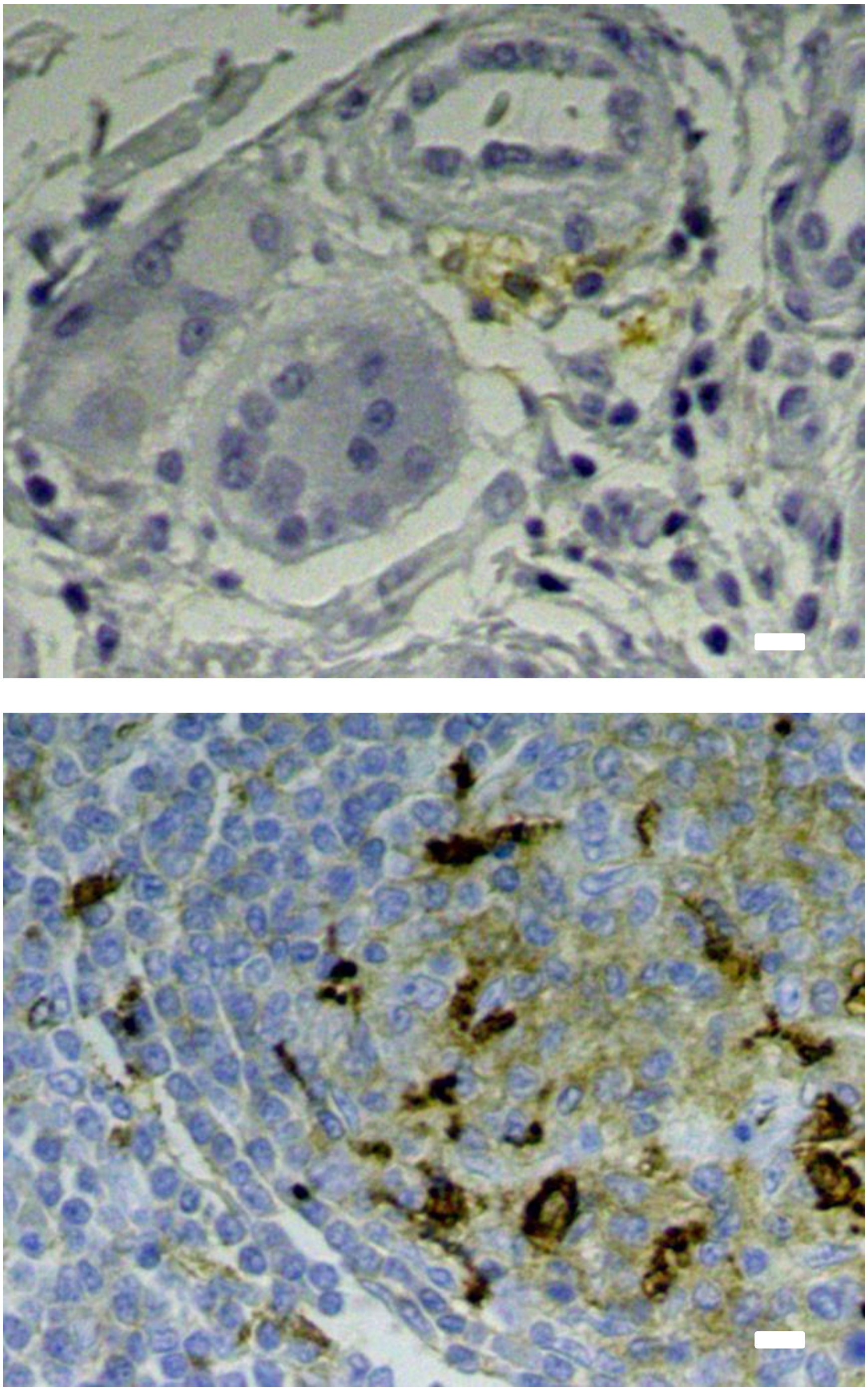

Dendritic cells DEC205 / Hygroma

DEC205 + control / Spleen

Supplemental Figure 1G

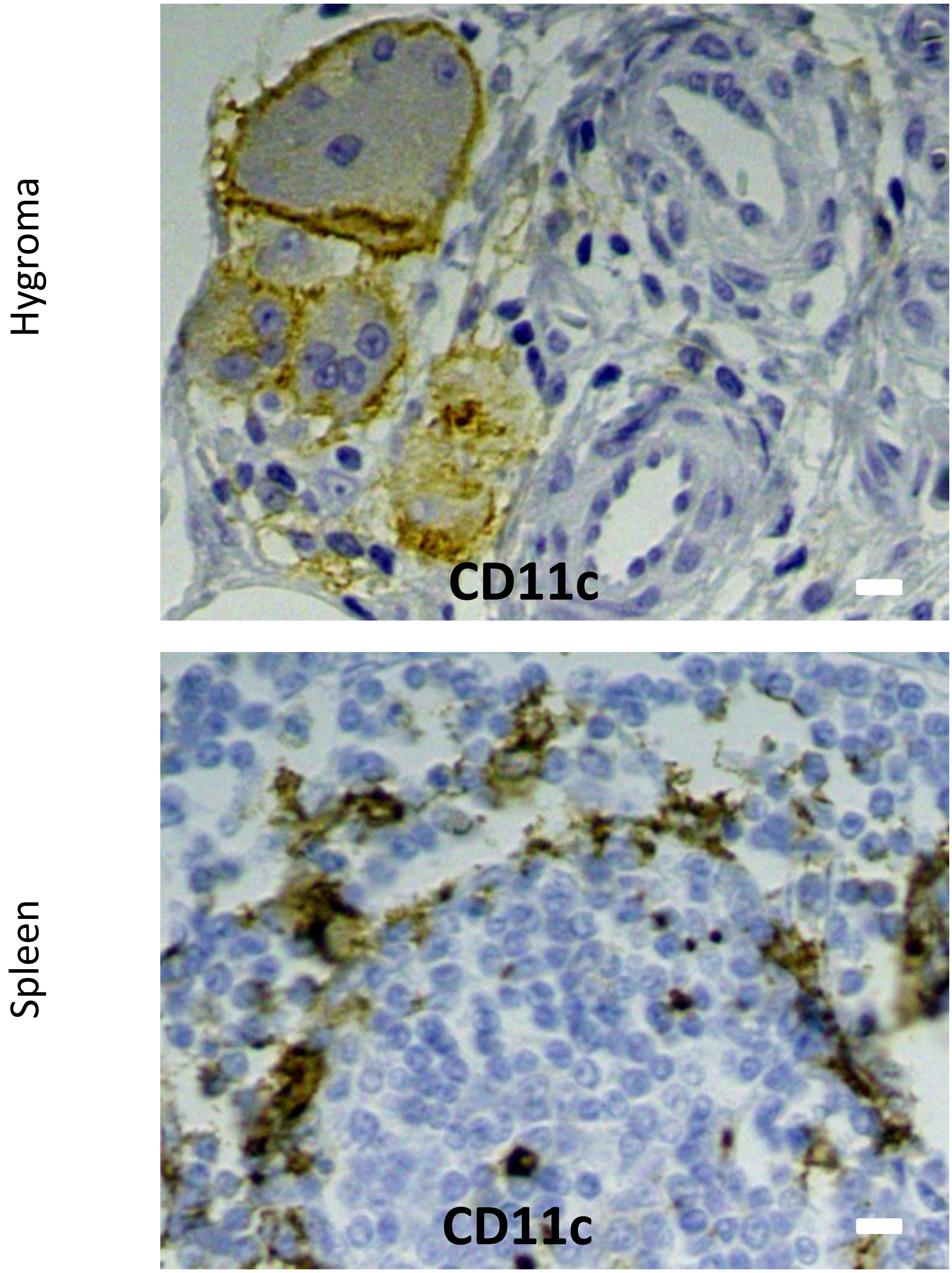

Macrophage CD11c+ / hygroma
CD11c+ control / Spleen
