# Supplementary figures and images for: Robust COX-2-mediated prostaglandin response may drive arthralgia and bone destruction in patients with chronic inflammation post-chikungunya
Source: PLoS Negl Trop Dis. 2021 Feb 17;15(2):e0009115. doi: 10.1371/journal.pntd.0009115 (PMC7920362; doi:10.1371/journal.pntd.0009115)

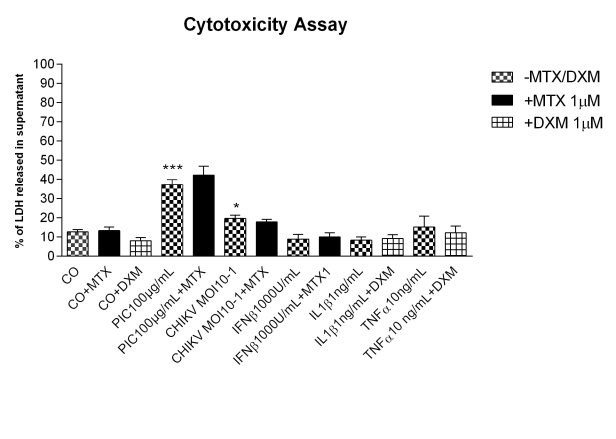

Supplement: S2 Fig — A) HSF were incubated with PIC 100μg/mL, CHIKV MOI 10–1, IFNβ 1000U/mL, IL1-β 1ng/mL and TNFα 10ng/mL in the presence or not of MTX 1μM or DXM 1μM for 24h. Cytotoxicity was monitored by measuring percentage of LDH released in culture supernatants. Results are from 3 independent experiments. ***p < 0.01 vs the control, *p < 0.05 vs the control, by one-way ANOVA followed by the Bonferroni’s test. (TIFF) [file pntd.0009115.s002.tiff]
